# Assessing Public Interest in Mpox via Google Trends, YouTube, and TikTok

**DOI:** 10.2196/48827

**Published:** 2023-09-06

**Authors:** Nicholas Comeau, Alyssa Abdelnour, Kurt Ashack

**Affiliations:** 1 Michigan State University College of Human Medicine Grand Rapids, MI United States

**Keywords:** monkeypox, Mpox, social media, internet, Google Trends, YouTube, TikTok, dermatologists, dermatology, dermatologist, awareness, interest, infectious, sexually transmitted disease, STD, sexually transmitted infection, STI, sexual transmission, sexually transmitted, outbreak, outbreaks, information seeking, information behaviour, information behavior, search, information quality, communicable

## Abstract

Public response to the recent Mpox outbreak was analyzed using internet search trends and social media posts.

## Introduction

With the advent of Mpox (formerly known as monkeypox), it is crucial for patients to better understand its symptoms and dermatological presentations. Social media platforms are accessible sources that can enhance disease awareness and knowledge of treatment options [1]. Unfortunately, video content on social media is not screened prior to dissemination, so misinformation on Mpox has increased tremendously. Content created by physicians is needed to increase awareness of the disease and its treatment options. We sought to evaluate the quality of social media posts related to Mpox on TikTok and YouTube.

## Methods

We used Google Trends to search for the term “monkeypox” to assess recent changes in searches between May and July 2022. YouTube and TikTok searches were performed with the term “monkeypox.” Results were evaluated for the presence or absence of a physician, and videos were assessed using DISCERN criteria. The Student *t* test was used to compare mean DISCERN scores between physician and nonphysician creators. DISCERN is a tool that is useful for evaluating consumer health information [2]. Previous studies have shown it is also useful in determining the quality of social media posts on health-related topics [3]. Only the first 50 videos on YouTube and TikTok were analyzed in order to replicate the general audience viewing experience. Videos not in English, videos without words, and duplicate videos were excluded.

## Results

We found that Google searches for the term “monkeypox” correlated with the prevalence of cases by state using Google Trends and data from the Centers for Disease Control and Prevention (Pearson *r*=0.74, *P*<.001) (Multimedia Appendix 1). Of the 50 TikTok videos analyzed (Table 1), 32 (64%) videos featured nonphysicians and 18 (36%) featured physicians. Videos featuring nonphysicians had an average DISCERN score of 1.82 (SD 0.44) whereas physician-created videos had an average score of 2.56 (SD 0.57) (*P*<.001).

**Table 1:** Overview of Mpox content on TikTok.

		Videos, n	Number of views, mean (SD)	Number of likes, mean (SD)	Number of comments, mean (SD)	DISCERN score, mean (SD)
**Content creator**						
	Physician	18	432,800 (503,684)	32,529 (53,659)	1038.83 (1518)	2.56 (0.57)
	Nonphysician	32	1,578,069 (2,209,723)	169,024 (307,217)	2286 (3260)	1.82 (0.44)
**Video type**						
	Educational	24	599,654 (1,091,832)	41,193 (74,693)	935.33 (4176)	2.41 (0.55)
	News	16	1,995,819 (2,751,170)	258,969 (409,353)	3352 (4176)	1.82 (0.29)
	Personal testimony	10	1,196,380 (1,111,310)	86,214 (93,152)	1576.80 (1656)	1.77 (0.72)

Our analysis revealed that physician-created YouTube videos had a mean DISCERN score of 3.31 (SD 1.15), while nonphysician videos had a mean score of 1.99 (SD 0.36) (Table 2). However, the difference between the DISCERN scores was not statistically significant (*P*=.35). Of the 50 YouTube videos evaluated, 37 videos featured nonphysicians describing the outbreak in the United States, while only 2 videos were created solely by physicians, which likely caused limitations in the DISCERN analysis. A total of 11 videos were excluded from the analysis: 2 were duplicate videos, 1 was a YouTube “short,” and 8 videos were created by news sources that featured physician speakers.

**Table 2:** Overview of Mpox content on YouTube.

Content Creator	Videos, n	Number of views, mean (SD)	Number of likes, mean (SD)	Number of comments, mean (SD)	DISCERN score, mean (SD)
Physician^a^	2	250,803 (138,908)	7100 (3652)	980 (550)	3.31 (1.15)
Nonphysician^b^	37	27,066 (83,888)	361 (1144)	304.73 (507)	1.99 (0.36)

^a^The video type was “educational” for both videos.

^b^The video type was “news” for all videos.

## Discussion

### Principal Findings

Based on the Google Trends analysis, there was an increase in public interest for Mpox, which occurred during the disease outbreak. This shows an increase in community response to the Mpox outbreak by searching for additional information via Google.

Analysis of videos on YouTube and TikTok identified a need for physician-created content to provide quality educational information on Mpox. With increased social media usage by physicians, these platforms can be used as an educational tool while also decreasing the spread of both infection and misinformation.

Physician-created TikTok videos had a significantly higher DISCERN score, indicating a higher quality of consumer health information. Physicians scored particularly well on the questions “is it balanced and unbiased?” and “does it describe how each treatment works?”. The majority of YouTube videos found were created by news sources, and the difference in DISCERN averages between nonphysician and physician data was not significant. A limitation of this study is the inclusion of only a small number of physician-created videos in the analysis.

### Conclusion

To summarize, Google Trends remains a useful tool for analyzing the public response to local disease outbreaks [4]. This could hold future potential in monitoring the spread of disease even before statistical data indicate a local outbreak. Additional research is needed to investigate whether a temporal relationship between Google searches and local disease outbreaks exists.

In general, the quality of videos on both TikTok and YouTube can be improved if content creators discuss the risks and benefits of treatments, provide references for the information shared in their videos, and collaborate with physicians.

## Appendix

Multimedia Appendix 1 Cases reported by the Centers for Disease Control and Prevention from May 1, 2022, to July 26, 2022 (left) and “monkeypox” searches by state from the same period (right).
