# Supplementary figures and images for: Assessing Public Interest in Mpox via Google Trends, YouTube, and TikTok
Source: JMIR Dermatol. 2023 Sep 6;6:e48827. doi: 10.2196/48827 (PMC10512109; doi:10.2196/48827)

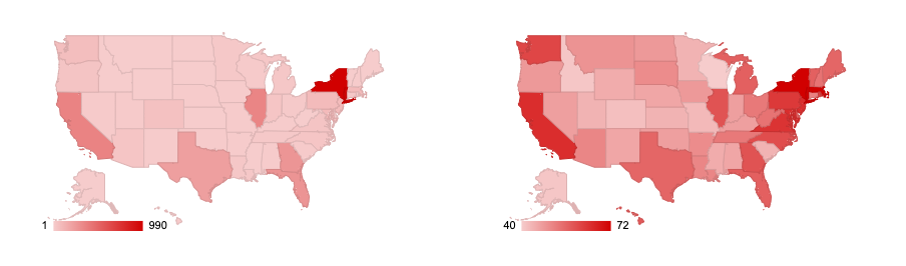

Supplement: Multimedia Appendix 1 [file derma_v6i1e48827_app1.png]
